# Triboostcardio ensemble model for cardiovascular disease detection using advanced blockchain-enabled health monitoring

**DOI:** 10.3389/frai.2025.1734013

**Published:** 2026-01-16

**Authors:** M. Mayuranathan, V. Anitha, P. Nehru, Bosko Nikolic, Miloš Janjić, Nebojsa Bacanin

**Affiliations:** 1 Department of Computer Science and Engineering, SRM Valliammai Engineering College, Chengalpattu, Tamil Nadu, India; 2 Department of Computer Science and Engineering, Panimalar Engineering College, Chennai, Tamil Nadu, India; 3 Department of Computer Science and Engineering, B. S. Abdur Rahman Crescent Institute of Science and Technology, Chennai, Tamil Nadu, India; 4 School of Electrical Engineering, University of Belgrade, Belgrade, Serbia; 5 Innovation Centre, School of Electrical Engineering, University of Belgrade, Belgrade, Serbia; 6 Faculty of Informatics and Computing, Singidunum University, Danijelova, Belgrade, Serbia; 7 Department of Mathematics, Saveetha School of Engineering, SIMATS, Thandalam, Chennai, Tamil Nadu, India

**Keywords:** blockchain, cardiovascular disease, detection, healthcare, optimization, patient monitoring

## Abstract

**Introduction:**

Heart diseases (CVDs) are a major cause of morbidity and mortality in all global regions and thus there is the pressing need to develop early detection and effective management approaches. Traditional cardiovascular monitoring systems do not necessarily have real-time analyzing solutions and individual understanding, which leads to delayed interventions. Moreover, one of the greatest issues in digital healthcare applications remains to be data privacy and security.

**Methods:**

The proposed research is to present a developed model of CVD detection that will combine Internet of Things (IoT)-based wearable devices, electronic clinical records, and access control using blockchain. The system starts by registering patients and medical personnel and then proceeds with collecting physiological as well as clinical data. Kalman filtering helps in improving data reliability in the pre-processing stage. Shallow and deep feature extraction methods are used to describe complicated patterns of data. A Refracted Sand Cat Swarm Optimization (SCSO) algorithm is used as part of feature maximization. A new TriBoostCardio Ensemble model (CatBoost, AdaBoost, and LogitBoost) is used to conduct the classification task and enhance the predictive accuracy. Smart contracts provide safe and transparent access to health information.

**Results:**

There are experimental results that the proposed framework enhances high predictive accuracy and detecting cardiovascular diseases earlier than traditional ones. The combination between SCSO feature selection and the TriBoostCardio Ensemble model improves the sturdiness of the model and precision of classification.

**Discussion:**

Besides the fact that the presented framework promotes the accuracy and timeliness of CVD detection, it also way to deal with important problems related to the data privacy and integrity with the help of blockchain-based access control. This solution offers a stable and trustworthy solution to the current healthcare systems with the combination of the smart optimization of features, ensemble learning, and secure data management.

## 1 Introduction

Cardiovascular diseases (CVD) continue to be a significant global health issue, requiring new and creative strategies to address the shortcomings of conventional healthcare methods. Given the annual high number of deaths due to CVDs, there is a clear requirement for ongoing monitoring and early identification (Moshawrab et al., 2023; Chang et al., 2022; Nagavelli et al., 2022). The rise of the Internet of Things brings about a revolutionary method by connecting wearable devices and sensors to offer immediate health information (Keikhosrokiani and Kamaruddin, 2022). This move toward constant, individualized monitoring signifies a pivotal advancement in cardiovascular care, filling in the deficiencies in standard occasional clinical methods (Saeed et al., 2023; Ahmed et al., 2022).

In the changing field of healthcare, advanced technologies have led to new solutions for addressing important challenges. One such significant approach is combining Internet of Things and blockchain technologies for monitoring cardiovascular disease in remote patients (Alshamrani, 2022). This innovative combination improves healthcare efficiency and ensures the security of patient data. Cardiovascular diseases remain a leading cause of death globally, highlighting the need for continuous monitoring to detect early signs and prevent adverse events. Traditional healthcare models often lack real-time insights into patients’ heart health, prompting the exploration of advanced technologies to fill this gap (Eisa and Alnaggar, 2022; Tiwari et al., 2022; Ketu and Mishra, 2022). The synergy of IoT and blockchain presents a promising solution to overcome these limitations in conventional methods.

The Internet of Things makes it possible to connect medical devices, wearables, and sensors, allowing vital signs to be monitored continuously in real time. This interconnected system gives healthcare providers access to a complete set of data that provides patient’s cardiovascular health data without clinical visits (Hasanova et al., 2022; Roy et al., 2022). Wearable devices with embedded sensors track physiological measurements like heart rate, blood pressure, and ECG data and send this information securely to a central system.

Blockchain technology strengthens the healthcare system by addressing issues of data security, privacy, and integrity. Its decentralized and unchangeable nature ensures that patient data cannot be altered and is only accessible to authorized individuals. This creates trust among patients and healthcare providers, promoting a transparent and secure environment for managing sensitive health information (Dammak et al., 2022; Bataineh et al., 2022; Azbeg et al., 2022).

The combination of IoT and blockchain in remote patient monitoring for detecting cardiovascular disease is driven by a comprehensive effort to improve healthcare results. This method seeks to give patients more control through ongoing monitoring, promoting a model centered on the patient. At the same time, it deals with important issues like data security and privacy by using blockchain’s decentralized and tamper-resistant characteristics. By encouraging early detection, personalized care, and cost-effective treatments, this integration signifies a crucial move toward a future in healthcare defined by proactive strategies that involve patients and better overall health results.

- To develop a real-time patient monitoring system using the combination of IoT and Blockchain in healthcare and privacy and trust.
- The use of RL-SCSO for feature selection improves convergence toward an optimal subset essential for robust CVD detection.
- The proposed TriBoostCardio Ensemble Model uses CatBoost, AdaBoost, and LogitBoost for detecting CVD which outruns the existing methods.

This paper is structured as follows: Section 2 reviews the literature that highlights the worldwide incursion caused by cardiovascular diseases (CVDs), the drawbacks of traditional monitoring systems, and the opportunities of the IoT and blockchain technologies in terms of continuous, safe, and real-time health surveillance. Section 3 outlines the materials used and the methodology proposed to be used in the detection of cardiovascular disease. Sections 4, 5 cover the results of the experiment with comparative analyses of the current methods. Lastly, Section 6 summarizes the major findings of the paper, gives recommendations, enumerates limitations, and proposes future research directions.

## 2 Related works

This section presents the Recent research on cardiovascular disease (CVD) detection has grown to prioritize the use of machine learning, deep learning, and IoT-based monitoring systems to enhance the accuracy of the diagnostic and real-time analysis. Hybrid and ensemble models, feature optimization methods and signal-based methods that utilize ECG and PPG signals have been studied. Nevertheless, most of the available approaches have issues of interpretability, scalability, and data security. To overcome these shortcomings, the latest literature has started to use blockchain and sophisticated optimization models to boost the model transparency, performance, and reliability in healthcare applications.

Konstantonis et al. (2022) analyzed the detection of cardiovascular disease in rheumatoid arthritis patient’s carotid/femoral arterial imaging and using machine learning methods. The study categorized CVD risk factors into office-based measures, carotid ultrasound image-based phenotypes, and blood biomarkers. Three machine learning classifiers such as Linear Discriminant Analysis (LDA), Support Vector Machine (SVM), and Random Forest were utilized. The study analyzed CVD risk factors in three categories: carotid ultrasound phenotypes, blood biomarkers, and conventional measures. The study assessed the performance of these classifiers to predict CVD risk in rheumatoid arthritis patients. While the research contributes to the intersection of machine learning and cardiovascular health, more specific performance metrics and clinical implications would enhance its impact.

Kallimani et al. (2022) presented a new method, Hybrid Deep Learning-Based Heart Disease Detection and Classification (FSHDL-HDDC) technique, for detecting and classifying heart disease in e-healthcare. It combines data normalization, missing value imputation, and the elite opposition-based squirrel search algorithm (EO-SSA) algorithm for feature selection. The addition of ACNN-LSTM improves heart disease detection. While this model demonstrates innovative feature selection using EO-SSA and powerful deep learning techniques, its complexity could make it challenging to interpret and computationally inefficient. Balancing model complexity with practical deployment is vital for integrating it into real-world e-healthcare environments seamlessly.

Alqahtani et al. (2022) presented a method that combines machine learning and deep learning models to predict cardiovascular disease. The approach achieves an accuracy of 88.70% by using six classification algorithms and random forest for feature extraction from a publicly available dataset. Although the ensemble approach improves prediction accuracy, it may lack transparency in decision-making due to its combination of various algorithms. A more thorough examination of the model’s interpretability could enhance understanding of its practical use and challenges in clinical settings.

De Vries et al. (2023) examined the use of non-invasive electrocardiography with artificial intelligence to detect congenital heart disease in fetuses. The study involves training an artificial neural network (ANN) on fetal electrocardiograms and incorporating a Bayesian updating rule to improve its performance. While the study provides valuable insights into non-invasive CHD detection, it lacks information about the interpretability of the algorithm and potential false-positive/negative rates. The opaque nature of artificial neural networks could affect clinical trust, underscoring the need for further investigation into their transparency for successful integration into prenatal care practices.

Al Bataineh and Manacek (2022) improved the heart disease prediction by using various machine learning (ML) algorithms, with a focus on a multilayer perceptron trained with a particle swarm optimization (PSO) algorithm. The study compares 10 different ML algorithms on the Cleveland Heart Disease dataset. Hybrid models can be complex and may pose challenges in understanding decision-making processes. A more thorough exploration of the algorithm’s transparency and its potential limitations in real-world clinical applications could enhance the evaluation of the MLP-PSO algorithm.

Islam et al. (2023) presented an Internet of Things system for remote health monitoring. This system uses sensors to measure heart rate, blood oxygen level, ECG signal data, and body temperature. The aggregated data is sent to a server by executing the MQTT protocol. Finally, the CNN architecture with an attention layer classifies potential diseases. While this system provides comprehensive real-time health monitoring, it may lack clarity in explaining how the deep learning model makes decisions due to its complexity. This could raise concerns about trusting the accuracy of the system’s diagnostic outcomes and gaining trust in the system’s recommendations for successful implementation in real-world healthcare scenarios.

Sadad et al. (2022) introduced a new method for detecting cardiovascular disease using photoplethysmography signals with IoT-enabled wearable patient monitoring devices. The research investigates machine learning methods such as decision tree, naive Bayes, and SVM along with one-dimensional CNN-long short-term memory (1D CNN-LSTM). The system, designed for continuous monitoring, achieves an impressive accuracy of 99.5% using the PPG-BP dataset. Cloud computing is used to improve the efficiency and connectivity of the monitoring system for cardiac patients. However, more attention should be given to ensuring that the proposed model can be applied effectively across different patient groups.

Samuel et al. (2023) developed a blockchain-based coalition network to share COVID-19 information securely. In the developed coalition system, health facilities can exchange information while optimizing their profits. Furthermore, each organization selected the finest replies from the suggested fictional play to study other people’s techniques and update its own beliefs. From the investigation outputs, it exposed 15% minimized computational cost and 26% proof-of-work.

Rani et al. (2022) suggested a health monitoring system using various CNN models. Here, blockchain was used to enable security. The suggested routing method routed the data to its destination with the least amount of energy consumption and network overhead by taking into account variables like likelihood, credibility rating, and node energy. The simulation results exposed better performance by means of 92% accuracy.

## 3 Materials and methods

In this section, the proposed framework, which is the combination of wearable IoT-powered sensors, electronic clinical records, and blockchain technology, to offer the secure and reliable cardiovascular disease detection, is introduced. Patients and the staff members are registered with the help of smart contracts, which offer access to any data in a specified manner and can be audited. Kalman filtering eliminates disturbance and inaccuracy in the wearable sensor-based physiological parameters. It is based on a combination of shallow and deep features representations to examine the input image in their entirety, and the Refracted Sand Cat Swarm Optimization (SCSO) algorithm to apply the most useful features in selecting the best ones. TriBoostCardio Ensemble Model as the combination of CatBoost, AdaBoost and LogitBoost can achieve the classification accuracy improvement but blockchain-based access control ensures the data safety, preservation of transparency and privacy.

**Figure 1:**
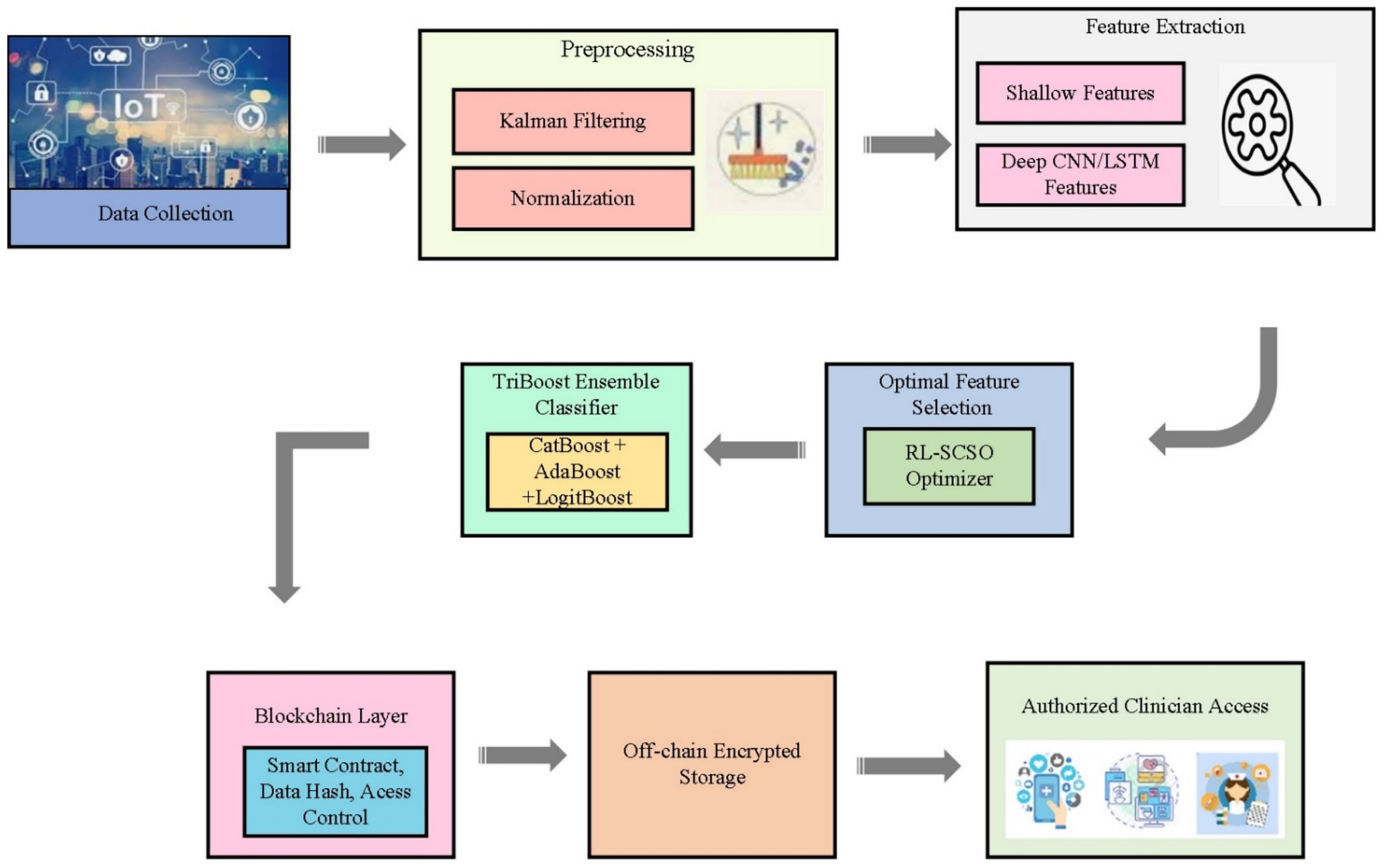
End-to-end system architecture of the proposed TriBoost Cardio framework.

The system architecture is a general system that incorporates the IoT-based data acquisition, preprocessing, feature engineering, feature optimization, classification, and blockchain-secured storage displays in Figure 1. IoT edge devices initially gather physiological indications and clinical measurements and send them to the gateway. Kalman filtering is used to de-noise the sensor readings and stabilize them and then the normalization and feature construction is performed. CNN/LSTM models are used to extract shallow clinical features and deep representations and combine them into a single feature vector. The RL-SCSO optimizer then picks the most discriminative features and these are inputted into the TriBoost ensemble classifier comprising of CatBoost, AdaBoost, and LogitBoost classifiers. The resulting CVD prediction and the data hash are uploaded to the Ethereum blockchain via a smart contract and the entire encrypted record is stored off-chain. The stored results are accessed by authorized medical personnel through permission verification on-chain.

### 3.1 Registration

In the proposed system for monitoring and detecting heart disease using blockchain technology, a registration process is done for both patients and medical staff to ensure efficient data management and access control.

#### 3.1.1 Patient’s registration

The Medical healthcare service provider (MHSP) registers the details of all patients receiving hospital services, including those who are hospitalized as well as elderly individuals being remotely monitored by medical staff. Patient details, such as their Ethereum address used as phone number, age, name, ID, etc., are securely stored in the blockchain. Each patient is assigned to a specific Medical Staff member who oversees their healthcare needs. Patients receive authorization to access and review their health data.

#### 3.1.2 Staff registration

**Figure 2:**
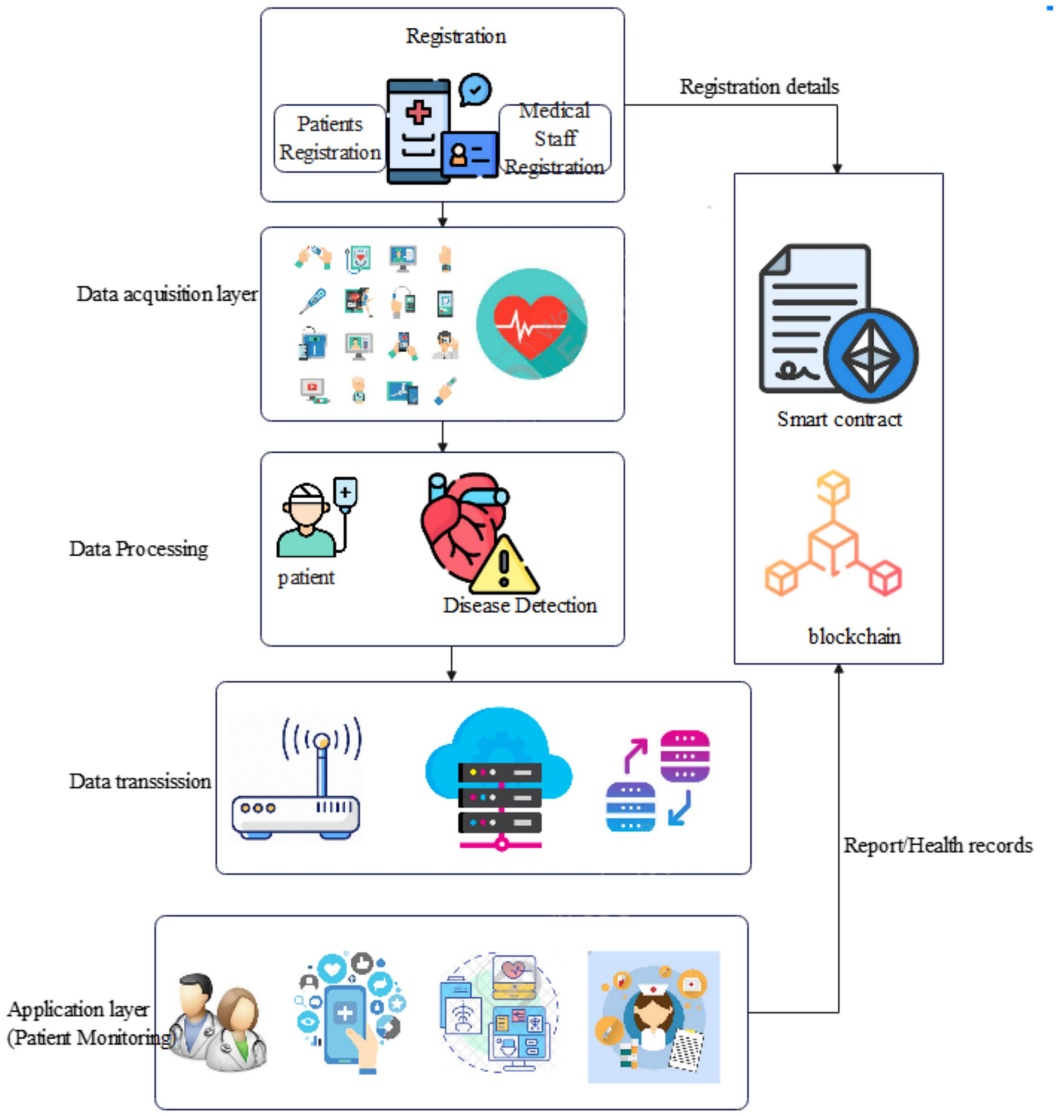
Architecture of the proposed health monitoring system.

The registration process also includes Medical Staff, which consists of doctors, nurses, and paramedical staff. Each member is registered by the MHSP with personal details such as an Ethereum address for identification, name, license ID, and role. It is important to ensure that the appropriate permissions are given to each medical staff within this access control system. Doctors can access historical patient data, request real-time information, and create reports for diagnosis and treatment. Nurses have access to both historical and real-time patient data and can request doctor interventions in critical conditions. Paramedical staff can process requests related to specific patients like meal preparation, patient transfers, and sample collections for laboratory analysis. The patient’s ID is used to display relevant parameters for the requested medical staff members in order to maintain secure data-sharing environment within the blockchain-based healthcare system. Architecture of the proposed health monitoring system shown in Figure 2.

### 3.2 Data acquisition

The dataset used in the study is the publicly available CVD in Kaggle in the links https://www.kaggle.com/datasets/jocelyndumlao/cardiovascular-disease-dataset and https://physionet.org/content/scg-rhc-wearable-database/1.0.0/ [Access Date 01-07-2024], which has 70,000 patient records and 14 clinical attributes. The data set is composed of demographic data (age, gender), variables of symptoms (type of chest pain), physiological variables (resting blood pressure, serum cholesterol, maximum heart rate, ST depression), and diagnostic variables (fasting blood sugar, resting ECG, exercise-induced angina, slope of the ST segment, and number of major vessels). The target variable is a binary variable, that is, the absence (0) or presence (1) of cardiovascular disease. Attributes are both numeric and categorical with ranges of resting BP (94–200 mmHg), serum cholesterol (126–564 mg/dL), maximum heart rate (71–202 bpm), and oldpeak (0–6.2). The data provides a wide range of patient features, which develop a strong model. Data variability, inconsistency management, and bias due to an imbalance were to be prevented by doing descriptive statistics and distribution checks. The study uses a publicly available, fully anonymized cardiovascular disease dataset obtained from Kaggle. No personal identifiers are included, and no new human data were collected. As the analysis is conducted on secondary de-identified data, ethical approval and informed consent are not required.

### 3.3 Data pre-processing

The Data Pre-Processing Layer is important for ensuring the accuracy of heart disease prediction from wearable sensor data. These data are often inconsistent, incomplete, and noisy. To handle these challenges, various pre-processing techniques are used. One of these techniques is Kalman filtering, which effectively removes duplicate records, noise, and discrepancies from the data. This unsupervised filtering algorithm works well with real-time sensor data by providing values closer to the actual sensor readings without adding extra noise. Additionally, two other unsupervised filters are used: one removes irrelevant attributes while the other replaces missing values with mean or median values to improve the overall quality of the structured dataset.

Traditional methods for storing data, such as off-chain servers or cloud/Blockchain platforms, encounter challenges related to unauthorized changes or deletions and high costs. This proposed architecture suggests a dynamic approach to data storage. Instead of saving all retrieved data without discrimination, each piece of information undergoes analysis and is compared to predefined limits. The values that go beyond these thresholds are stored in the secure and tamper-resistant Blockchain platform. This helps reduce the risk of data manipulation and maximize resource efficiency.

**Figure 3:**
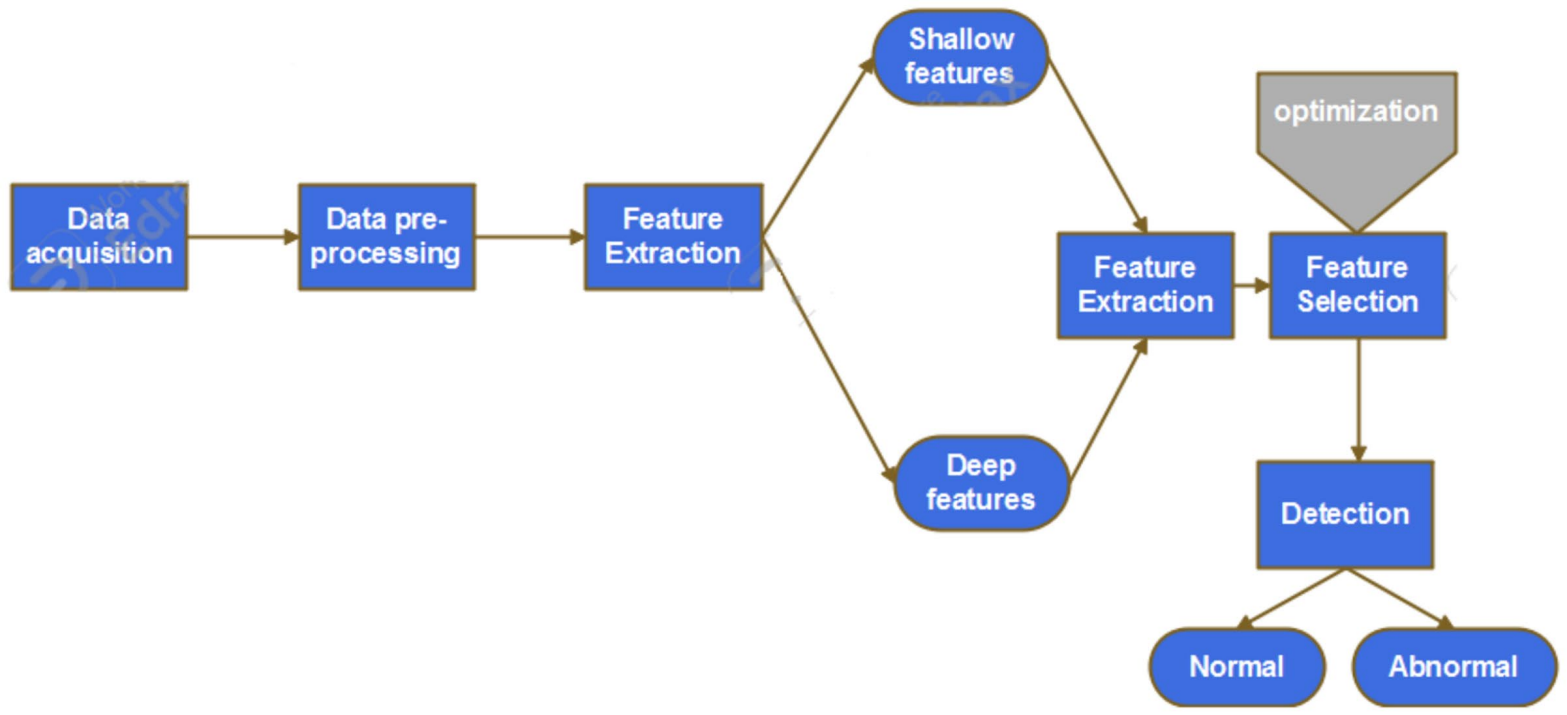
Block diagram of the proposed disease detection framework.

Heart Rate Analysis evaluate the heart rate data obtained from a sensor in relation to the patient’s age retrieved from the Blockchain using their unique patient ID. The algorithm uses a function named heart_rate that returns a boolean value indicating whether the heart rate falls within acceptable limits. Abnormal heart rate patterns are flagged based on specified conditions within this function. By dynamically determining thresholds based on the patient’s age, it ensures an age-specific assessment of heart rate. If the heart rate exceeds or falls below the thresholds, it signals potential deviation requiring further attention or intervention by setting the result as True; otherwise, it remains False. This provides real-time monitoring of anomalous heart rates for prompt identification and addressing of potential health concerns in patients by healthcare professionals. Figure 3 displays Block diagram of the proposed disease detection framework.

This adaptive strategy ensures that only pertinent and substantial data exceeding predetermined benchmarks is retained, thereby contributing to efficient use of storage resources and robustness in predicting heart disease within this proposed framework. Kalman filtering improves the accuracy of the physiological data that is sent by wearable IoT sensors by minimizing noise, removing measurement anomalies, and fixing gaps or unreliable measurements. IoT devices are often subject to noise caused by motion artifacts, signal interference as well as environmental changes. Kalman filter is a recursive estimator that forecasts the next state of the physiological measurement and corrects it with sensor data that are observed. This prediction–correction process smooths signal variations, detects anomalous values, and estimates corrupted values with statistically optimal values. Kalman filtering enhances downstream feature extraction, classification accuracy, and guarantees reliable data to monitor real-time cardiovascular disease due to the provision of cleaner and more stable input signals.

### 3.4 Feature extraction

#### 3.4.1 Shallow features

##### 3.4.1.1 Heart rate

The heart rate is a key physiological measure indicating the number of heartbeats per minute (bpm). It can be measured by feeling peripheral arteries or using medical devices like electrocardiograms. The normal resting heart rate for adults usually ranges from 60 to 100 bpm. When the heart consistently beats above 100 bpm, it may indicate tachycardia and signal conditions such as fever or cardiac issues. On the other hand, a consistent heart rate below 60 bpm, known as bradycardia, could point to conditions like heart block, hypothyroidism, or an athlete’s well-conditioned heart.

##### 3.4.1.2 Blood pressure

It is essential for evaluating cardiovascular health and provide important information about the functioning of the circulatory system. Systolic and diastolic blood pressure, measured in millimeters of mercury (mmHg), indicate the force exerted by the heart during contraction and at rest between beats, respectively. Elevated values in either category are linked to increased cardiovascular risk, highlighting the importance of maintaining optimal blood pressure levels for overall health. Pulse pressure, calculated as the difference between systolic and diastolic pressures, offers valuable insights into arterial stiffness and compliance, aiding in identifying potential cardiovascular issues. Monitoring these blood pressure features are vital components of preventing and managing cardiovascular disease, enabling timely actions to promote overall cardiovascular well-being.

##### 3.4.1.3 Lipid profile feature

It provide important information about the levels of various lipids and cholesterol components in the blood, offering insights into cardiovascular health. The lipid profile includes measurements of total cholesterol, low-density lipoprotein (LDL) cholesterol, high-density lipoprotein (HDL) cholesterol, and triglycerides.

##### 3.4.1.4 Total cholesterol

It represents the overall amount of cholesterol in the blood.

##### 3.4.1.5 Low-density lipoprotein

It is often labeled as “bad” because elevated levels can contribute to arterial plaque formation.

##### 3.4.1.6 High-density lipoprotein

It is considered “good” as it helps remove LDL from the bloodstream, reducing the risk of arterial blockages. Triglycerides indicate excess calories or unhealthy dietary habits. High levels of triglycerides and LDL, along with low HDL increase the risk of atherosclerosis and coronary artery disease.

##### 3.4.1.7 Body mass index features

It refers to measurements related to the size, shape, and composition of the human body. It estimates body fat based on weight and height. Excess weight may be linked to an increased likelihood of developing cardiovascular diseases such as hypertension, diabetes, and coronary artery disease.

#### 3.4.2 Deep features

##### 3.4.2.1 ECG signal

Convolutional Neural Networks are crucial in healthcare, particularly for cardiovascular disease detection. CNNs automatically learn hierarchical and spatial representations, making them effective in processing medical images and time-series data like electrocardiograms. 1D CNNs extract precise features from raw ECG signals by capturing local patterns within the temporal domain. This capability helps in identifying specific cardiac cycle components such as P-waves, QRS complexes, and T-waves. For medical imaging purposes, both 2D or 3D CNNs efficiently process angiograms or MRI scans without manual feature engineering. This ability is helps in identifying structural abnormalities within the cardiovascular system. It not only enhances the accuracy of diagnosis but also streamlines the analysis of intricate patterns within medical images and time-series data.

##### 3.4.2.2 Temporal patterns

We use RNNs to capture temporal patterns and long-term dependencies crucial for understanding heart conditions. RNNs, especially LSTMs, are designed to excel in understanding long-term connections within sequential data. In ECG analysis, these networks can comprehend the chronological relationship between successive heartbeats. LSTMs use memory cells to selectively store and remember information from previous time steps, enabling them to learn and recall patterns, irregularities, and variations in the ECG signal. The ability of LSTMs to capture sequential connections is essential for accurate detection and prediction of arrhythmia. By understanding the detailed relationships between consecutive heartbeats, these networks can identify abnormal patterns that indicate irregular heart rhythms.

#### 3.4.3 Feature fusion

Feature fusion combines shallow and deep features to create a more effective representation of physiological data. Shallow features offer insights into the temporal and statistical characteristics of signals, while deep features capture spatial and temporal patterns. The process involves combining these distinct feature sets for improved discriminative power, robustness, information utilization, adaptability to diverse data types, and reduced overfitting. This approach enhances accuracy and interpretability in cardiovascular health assessment.

The feature-fusion approach combines shallow physiological features and deep temporal–spatial features derived based on ECG and clinical signals. Interpretable clinical features like heart rate, cholesterol level, blood pressure and symptom-based features are shallow features, which are good baseline features to cardiovascular risks assessment. Morphological patterns, rhythm dynamics, and long-term temporal dependencies, which are not represented by shallow descriptors, are encoded in deep features, which are obtained with CNN and LSTM networks. Fusion process is a concatenation of normalized shallow features and final deep-feature embeddings, resulting in a single high-dimensional feature. This joint representation is better at discriminability, as it combines clinical knowledge with automatically discovered signal patterns to create a richer and more informative feature space to the RL-SCSO optimizer and TriBoost classifier.

### 3.5 Feature selection

To select the relvant features we propose Refracted SCSO optimization which based on the behavior of sand cats in their natural environment. Sand cats have a special skill to hear sounds that are lower than 2 kHz, which distinguishes them from domestic cats. They are well-suited to living in harsh desert conditions and have adapted by having fur-covered soles and palms for protection against extreme temperatures, making it difficult to track their footprints. This algorithm is based on the behavior of sand cats in their natural environment. Sand cats have a special skill to hear sounds that are lower than 2 kHz, which distinguishes them from domestic cats. They are well-suited to living in harsh desert conditions and have adapted by having fur-covered soles and palms for protection against extreme temperatures, making it difficult to track their footprints.

The sand cat has excellent hearing, especially its sensitivity to low-frequency sounds, which makes it an extraordinary animal. In challenging environments, sand cats hunt at night and rest underground during the day in order to find prey when the temperature is cooler.

The sand cat’s foraging and hunting behavior involves quickly finding prey on the ground, which serves as the basis for the SCSO algorithm. This optimization process mimics the initial steps of population initialization seen in natural foraging behavior of sand cats.

The fitness function outlined in Equation 1 is computed.


$$\mathrm{Fit}=\lambda \delta +\eta \frac{|S|}{|T_{f}|}$$ (1)

where δ signifies the classification error rate. Additionally, ∣S∣ denotes the size of the chosen subset, while $T_{f}$ represents the total number of features within the dataset. The parameters λ and η are utilized to indicate the significance of both classification accuracy and subset length. Notably, λ falls within the range [0, 1], while η=(1−λ) .

#### 3.5.1 Search for prey

During the exploration stage, each sand cat’s location is represented as *Q*. The algorithm takes advantage of sand cats’ exceptional hearing ability for detecting low frequencies below 2 kHz. In mathematical terms, Equation 2 defines the sensitivity range *V_S_* and Equation 3 determines a crucial factor *U* that regulates balance between exploration and exploitation capabilities in the algorithm. These mathematical expressions are essential for guiding the algorithm’s search for prey during its exploratory phase, mirroring the keen sensory perception of sand cats in their natural habitat.


$$V_{S}=\gamma -(\frac{\gamma \times I}{I_{\max}})$$ (2)

$$U=2\times \mathrm{rand}(0,1)\times V_{s}-V_{s}$$ (3)

Where y=2 , I and $I_{\max}$ represents the current iteration and maximum iteration number, respectively.

During the exploration phase, each sand cat randomly moves to a new position within its range of sensitivity. This random behavior improves the efficiency of the algorithm for both exploring and exploiting resources. To prevent getting stuck in a local best solution, a strategy that promotes diversity is used by assigning a different sensitivity range $V_{\text{range}}$ to each sand cat. As described in Equation 4, this personalized sensitivity range guarantees that each sand cat explores a unique area, leading to more thorough exploration of potential solutions and decreasing the chance of early convergence.


$$V_{\text{range}}=\mathrm{rand}(0,1)\times V_{S}$$ (4)

Each sand cat dynamically adjusts its position in pursuit of prey, guided by an intricate interplay between its individualized sensitivity range $V_{\text{range}}$ , optimal candidate position $Q_{\mathrm{ocp}}(t)$ , and current location $Q_{\mathrm{cur}}(t)$ . The mathematical formula governing this adaptive movement is described in Equation 5. This formula makes a strategic exploration strategy, enabling the sand cats to efficiently explore and exploit the solution space as they adapt their positions based on the interplay of these factors.


$$Q(t+1)=V_{\text{range}}\times (Q_{\mathrm{ocp}}(t)-Q_{\mathrm{cur}}(t)\times \mathrm{rand}(0,1))$$ (5)

#### 3.5.2 Attack prey (exploitation stage)

In the exploitation stage, Equation 6 quantifies the proximity between the prey and the sand cat, simulating the moment when the sand cat attacks. The sand cat’s sensitivity range is represented as a circular area, and its movement direction is determined by a random angle β selected using the Roulette Wheel algorithm. This random angle ranges from 0° to 360°, corresponding to a value between [−1, 1]. This approach allows the sand cat to move in various directions within their search space. Afterward, Equation 7 gives the predatory movement toward the prey, ensuring that the cat advances toward its hunting position.


$$Q_{\mathrm{sc}-\mathrm{pr}}=|Q_{\text{best}}(t)\times \mathrm{rand}(0,1)-\text{Qcur}(t)|$$ (6)

$$Q(t+1)=Q_{\text{best}}(t)-Q_{\mathrm{sc}-\mathrm{pr}}\times V_{\text{range}}\times \cos(\beta )$$ (7)

#### 3.5.3 Refraction learning

Light refraction occurs when light passes through the boundary between two different mediums, such as air and water, causing the light to change direction due to a difference in speed. The RL technique operates based on this principle of light refraction. We incorporate this method in SCSO algorithm which helps in discovering optimal solutions by enabling a wider exploration range and preventing the algorithm to trap in local optima. It achieves this by considering various potential solutions and enhancing the trade-off between exploring new possibilities and exploiting existing ones. Additionally, the algorithm can prioritize specific areas where favorable solutions are more likely to be found with faster convergence.

The inverse of global optima $T^{*}$ can be determined through the process of refraction learning using Equation 10.


$$T^{*}=(\mathrm{LB}+\mathrm{UB})/2+(\mathrm{LB}+\mathrm{UB})/(2\mathrm{l\delta})-T^{*}/(\mu )$$ (8)

The refraction index μ is computed using Equation 11.


$$\mu =\frac{\sin\theta_{1}}{\sin\theta_{2}}$$ (9)

$$\sin\theta_{1}=((\mathrm{LB}+\mathrm{UB})/2-T^{*})/k$$ (10)

$$\sin\theta_{2}=T\prime *-((\mathrm{LB}+\mathrm{UB})/2)/k\prime$$ (11)

where T signifies the point of incidence (initial candidate solution) while T′ represents the point of refraction (opposite candidate solution). The center point of the search interval (*LB*, *UB*) is denoted by *CP*. Additionally, *g* indicates the distance between *Y* and *C*, and k′ represents the distance between T′ and *CP*.

RL-SCSO algorithm is an improvement of the feature-selection performance of the original SCSO optimizer by improving the exploration and exploitation stages. Traditional SCSO makes use of sensitivity-based movement to find the best feature subsets, and can suffer premature convergence in high-dimensional biomedical data. The RL element proposes the use of a refraction-based learning scheme that creates a counter-solution to the existing candidate based on a refractive index based on feature-space boundaries. The mechanism enhances the search diversity and makes the algorithm explore unvisited areas of the feature space. Consequently, RL-SCSO minimizes the chance of local minima trapping and approaches feature subsets with greater discriminative power with more uniformity. The enhanced search behavior results in the improved selection of features, less redundancy, and the increased accuracy of classification to predict cardiovascular disease.

### 3.6 TriBoostCardio ensemble detection model

The proposed TriBoostCardio Ensemble Model uses the strengths of CatBoost, AdaBoost, and LogitBoost, which provides a strong and adaptable method for detecting cardiovascular disease. CatBoost is recognized for its ability to handle categorical features and effectively learn complex patterns in the data. AdaBoost trains weak learners sequentially with a focus on misclassified instances, adding flexibility in challenging data scenarios. LogitBoost uses iterative logistic regression to enhance the model’s predictive capabilities by prioritizing instances that need extra attention. By combining these different boosting algorithms, the ensemble model benefits from their unique learning strategies, ensuring comprehensive coverage of intricate patterns within physiological data. The combination of voting or weighted averaging optimally integrates predictions from each base learner into a unified and accurate model.

In our proposed TriBoostCardio Ensemble Model for identifying cardiovascular disease, a majority voting method is used to combine predictions. Each individual model makes its own prediction, and the final decision is made by choosing the class that receives the most votes. This approach gives equal importance to the predictions of CatBoost, AdaBoost, and LogitBoost. For example, if two out of these three models predict no presence of cardiovascular disease while one predicts its presence, “no presence” is chosen as the final combined prediction based on majority voting. This enhances accuracy in detecting cardiovascular disease.

#### 3.6.1 LogitBoost

It is a variation of the boosting algorithm intended for binary classification tasks. It works by progressively improving the model’s predictive abilities through training weak learners in sequence. The process starts with initializing a base learner, usually a simple model such as a decision stump, and assigning equal weights to each instance in the dataset. Subsequently, it iterates through the training process, focusing on instances that were misclassified in the previous round. During each iteration, a new weak learner (logistic regression model) is trained on the weighted dataset with higher emphasis given to misclassified instances. The weights are updated based on the misclassification error. The final model is an additive combination of all trained weak learners; each contributes proportionally based on its accuracy. LogitBoost adapts its learning strategy by giving more weight to challenging instances and hence can adjust according to data complexity. This adaptability combined with using diverse weak learners makes LogitBoost robust and valuable for tasks like cardiovascular disease detection where capturing intricate patterns in physiological data is vital for accurate diagnosis.

#### 3.6.2 CatBoost

It is a high-performing gradient boosting algorithm that is beneficial for detecting cardiovascular disease. It effectively processes categorical variables without requiring extensive preprocessing, which is particularly advantageous in the context of cardiovascular health datasets. The algorithm utilizes a robust symmetric tree learning approach to capture complex patterns and dependencies within the data. By iteratively building an ensemble of decision trees and implementing a depth-growth strategy to control model complexity, CatBoost addresses overfitting concerns. Additionally, its efficient handling of missing values contributes to the overall robustness of the model. Finally, predictions from individual trees are combined in the final ensemble, resulting in a powerful and accurate predictive tool for cardiovascular disease detection. With its adaptability to categorical features and resistance to overfitting, CatBoost proves valuable in constructing predictive models for health-related tasks involving mixed types of features commonly found in cardiovascular health datasets.

#### 3.6.3 AdaBoost

Adaptive Boosting, also known as AdaBoost, is a robust ensemble learning technique used in the detection of cardiovascular diseases. It works by training weak learners sequentially and placing emphasis on misclassified instances to improve predictive accuracy. The algorithm starts with initializing a base learner, such as a decision stump, and assigns equal weights to each instance in the dataset. In successive iterations, AdaBoost focuses on misclassified instances from the existing ensemble by assigning them higher weights for increased attention. Weak learners like decision trees are then trained using the weighted dataset in an iterative manner. The final model is a weighted combination of these weak learners, where each learner’s contribution is proportional to its accuracy. Additionally, its ability to combine diverse outputs from weak learners and resistance to overfitting contribute significantly toward building reliable predictive models for cardiovascular disease detection.

TriBoost ensemble combines CatBoost, AdaBoost and LogitBoost, which uses the learning advantages of each booster. CatBoost is very useful with categorical and heterogeneous tabular data, AdaBoost is very useful with misclassified samples by using adaptive weighting, and LogitBoost is very useful with probability calibration using iterative logistic regression. Although both of these models are effective in their own right, they exhibit varying error patterns, that is, they make errors in different parts of the data space. Majority voting to combine them minimizes model specific bias and variance resulting in a more stable and robust classifier. This complementary behavior allows TriBoost to capture linear, nonlinear and boundary level variations in cardiovascular data better than any individual classifier. As a result, the ensemble has better generalization, higher accuracy of detection and better performance in imbalanced or noisy data conditions.

### 3.7 Application layer

This layer acts as a specialized tool to enhance the management and monitoring of cardiovascular health. It provides personalized features and insights to meet the unique needs of individuals diagnosed with heart conditions. Patients can use the app to monitor important health metrics like blood pressure, heart rate, and medication adherence in real-time. The app also includes automated reminders for medication schedules and appointments, ensuring that patients adhere to their treatment plans. Continuous monitoring combined with trend analysis allows for early detection of any deviations from baseline health parameters. Additionally, the app enables secure communication between patients and healthcare providers, facilitating remote consultations, result-sharing, and timely interventions.

### 3.8 Access control

The proposed smart healthcare system uses blockchain technology and a smart contract on the Ethereum network to simplify and secure various aspects of patient care, treatment, and payment processes. The system involves key entities such as the Smart Healthcare System, Drug Store for Healthcare Services, patients, medical insurers, and a Research and Development Lab. The algorithm outlines how the smart contract governs these interactions.

The proposed smart contract for access control leverages Ethereum’s blockchain technology to govern and secure interactions among users in a decentralized manner. The contract, named introduces a structured approach to managing user roles and permissions within a blockchain-based ecosystem. Within this contract, user roles, namely ‘Patient’, ‘Doctor’, and ‘Administrator’, are clearly defined through an enumeration named ‘UserRole’. The ‘UserDetails’ struct has essential information about each user, including their Ethereum address, assigned role, and registration status.

To ensure secure access, the contract employs two modifiers: ‘onlyRegisteredUser’ and ‘onlyUserRole’. The former verifies whether a user is registered, while the latter ensures that the user possesses the required role for specific functionalities. These modifiers serve as safeguards, preventing unauthorized access to critical sections of the smart contract.

The algorithm provides functions such as ‘registerUser’ for users to register with the smart contract, specifying their desired roles. An administrator, identified as having the role of an ‘Administrator’, can utilize the ‘grantUserRole’ function to assign additional roles to users. The contract also includes a sample function, ‘restrictedFunction’, representing restricted functionality that can only be accessed by registered users with the role of a ‘Doctor’.

This access control algorithm establishes a foundation for decentralized and secure user management within a blockchain. It ensures that users are registered and assigned appropriate roles, enhancing transparency and accountability.

The smart contract begins in a state labeled “NotReady,” and important information like PatientID, patient name, and the IPFS hash of the Electronic Health Record are set during the contract’s creation. The algorithm utilizes Ethereum’s smart contract capabilities to manage critical data efficiently ensuring verification when needed. Additionally it keeps track of insurance company approvals using mappings with associated hash values which contributes to creating an auditable record of this process. In practical terms, a patient initiates this process triggering requests for approval by several systems. The events functions ensure that everything proceeds stepwise. This approach encourages transparency, security, and accountability.

The use of blockchain technology is included to provide secure, non-tamperable, and auditory management of sensitive patient data, gathered by IoT sensors and clinical records. Conventional centralized healthcare data systems are prone to unauthorized modification, single-point failures and privacy violation. Blockchain overcomes these challenges by storing access permissions, patient identifiers and data-sharing rules in a decentralized registry that is protected by cryptographic hashing. The smart contracts also create a high access control, where only authorized medical personnel can access or modify patient information. All transactions are time-stamped and immutable, which makes blockchain better in terms of data integrity, traceability, and the absence of the ability to manipulate data retrospectively. This secure infrastructure guarantees trust, transparency and confidentiality in all the steps of data transmission and analysis in a real-time cardiovascular monitoring setting. Algorithm 1 explains the Refracted SCSO for optimal feature selection.

**ALGORITHM 1:**
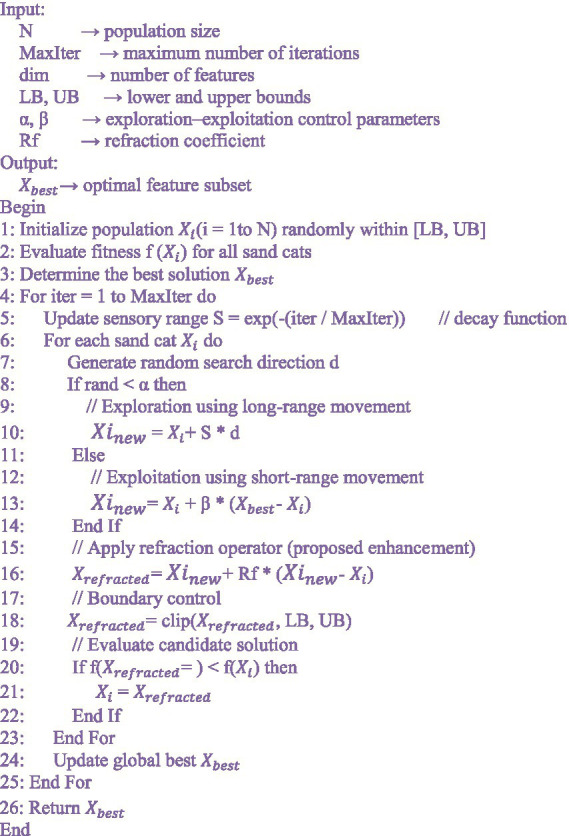
Refracted SCSO for optimal feature selection.

The elements incorporated into the suggested framework is chosen due to the fact that each of them solves a particular challenge of the IoT-based cardiovascular monitoring. The pre-processing requires Kalman filtering since the physiological signals of wearable devices are usually noisy, subject to motion artifact, and missing fluctuations; Kalman filtering offers real-time smoothing and precise determination of the state, which is trustworthy inputs to further analysis. Refracted SCSO is also added to the feature selection since cardiovascular datasets have redundant and correlated variables, which compromise the performance of classifier; SCSO has a good global search capability, converges efficiently, and avoids local optima better than the traditional optimizers, leading to a small and discriminative set of features. The TriBoostCardio ensemble (CatBoost, AdaBoost, and LogitBoost) are selected since each boosting algorithm has its own complementary advantages: CatBoost is better at using heterogeneous clinical data and categorical variables, AdaBoost is better at focusing on samples that are hard to classify, and LogitBoost is better at providing stable probabilistic modeling. The combination of these models produces superior robustness, better generalization and increased predictive accuracy compared to using each of the single classifiers individually. Combined, these elements guarantee an effective, noisy, and clinically stable CVD detection pipeline.

The suggested system include an Ethereum-based blockchain layer that guarantees the safety and impossibility to tamper with patient information. On-chain storage is only done on metadata, including the hashed patient ID, encrypted record pointer, and timestamp, with full clinical records stored in encrypted off-chain storage. IoT devices send hashed physiological data to a gateway, data is hashed and a transaction is sent to the blockchain using a lightweight smart contract based on role-based access control. Data is accessed by authorized clinicians by verifying on-chain permissions, and the off-chain encrypted file is retrieved. It is a hybrid on-chainarchitecture which facilitates integrity verification, decentralized auditing, and controlled data sharing at minimal blockchain storage and transaction cost.

In this paper, the blockchain block is deployed on a private Ethereum network with Ganache to provide low latency and complete control over data privacy. This network had 10 validator nodes who were operating under a lightweight PoA (Proof-of-Authority) consensus mechanism. The average gas cost was of 41,000 units, or approximately less than 0.002 ETH on a private chain. The mean transaction time is 1.2 s, and the block time was set to 2 s, which allows recording IoT data almost in real-time. This architecture guarantees scalability, low operation cost, and a simple way of dealing with continuous streams of physiological data.

## 4 Results

The proposed health monitor system for CVD detection using developed Triboost Cardio Ensemble model is implemented in MATLAB on Intel core® i5 processor, 2.6 GHz, 128 GB RAM, 64-bit OS. It is trained on CatBoost (500 trees), AdaBoost (200 estimators), LogitBoost (100 iterations), and RL-SCSO (population 100 iterations). Deep features are extracted with 1D-CNN and LSTM. Training is done on an 80:20 division, 5-fold cross-validation, Adam optimizer (LR = 0.001), batch size of 32 and Min-Max normalization with Kalman filtering. The blockchain layer is implemented on a private Ethereum (Ganache, PoA consensus) and the access-control verification of less than 10 ms. The experiment is carried out by using public access datasets from Kaggle. The data are collected from CVD dataset and patient data wearable Seismocardiogram Signal and Right Heart Catheter (SCG-RHC) devices from Kaggle. Also, the competence of the proposed model is verified via several simulation results with the existing models like ANN, SVM, RF, CatBoost, LogitBoost, and AdaBoost for various performance measures including accuracy, and sensitivity. Besides, the performance of the proposed RL-SCSO is verified via a comparative analysis with other optimization algorithms like COA, PSO, SSA, and MOA models.

### 4.1 Comparative evaluation

**Figure 4:**
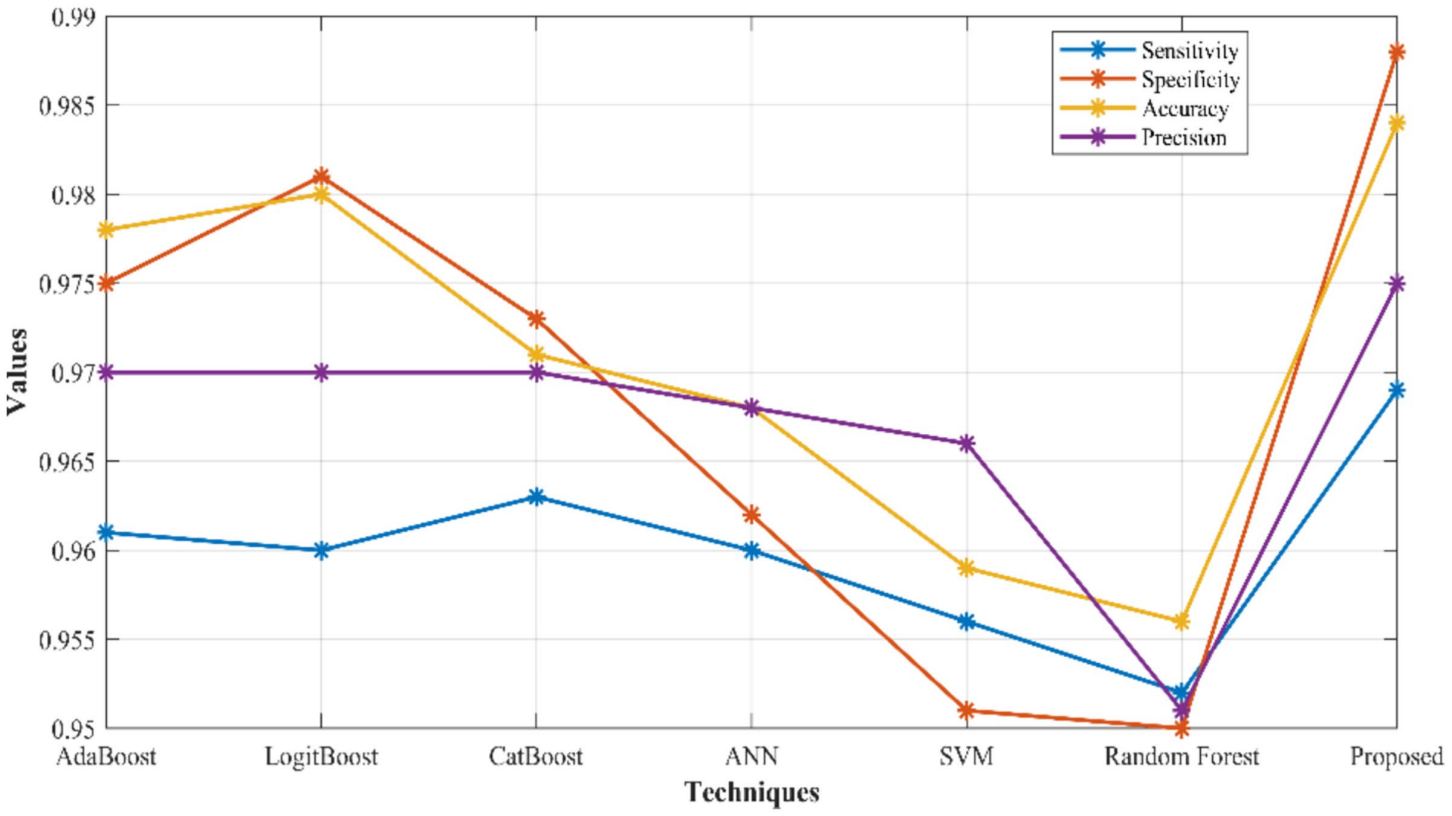
Comparative evaluation of suggested Triboost ensemble model over other models with respect to Acc, Sen, Spe, and Pre.

The proposed Triboost ensemble model and its attained results are discussed in this section. Here, the developed model accomplished better results as portrayed in Figure 4. For accuracy (Acc), the proposed network achieved 0.6% better than AdaBoost, 0.3% better than LogitBoost, 1.32% better than CatBoost, 1.62% improved than ANN, 2.54% better than SVM, and 2.84% improved than RF. Similarly, the proposed network attained 0.82, 0.84, 0.51, 0.84, 1.34, and 1.68% better than AdaBoost, LogitBoost, CatBoost, ANN, SVM and RF, respectively, for sensitivity (Sen). As for specificity (Spe), the introduced model reached enhanced results which is 1.31, 0.7, 1.51, 2.63, 3.74, and 3.84% better than AdaBoost, LogitBoost, CatBoost, ANN, SVM and RF, respectively. The developed Triboost ensemble model reached improved precision (Pre) results which is 0.51, 0.51, 0.51, 0.71, 0.92, and 2.46% better than AdaBoost, LogitBoost, CatBoost, ANN, SVM and RF, respectively.

**Figure 5:**
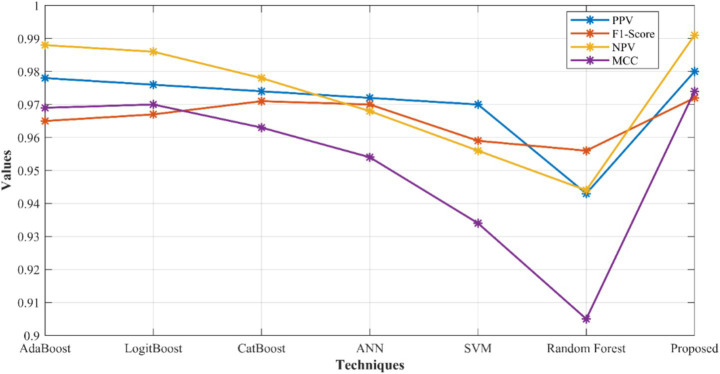
Comparative evaluation of suggested Triboost ensemble model over other models with respect to PPV, F1-score, NPV, and MCC.

**Figure 6:**
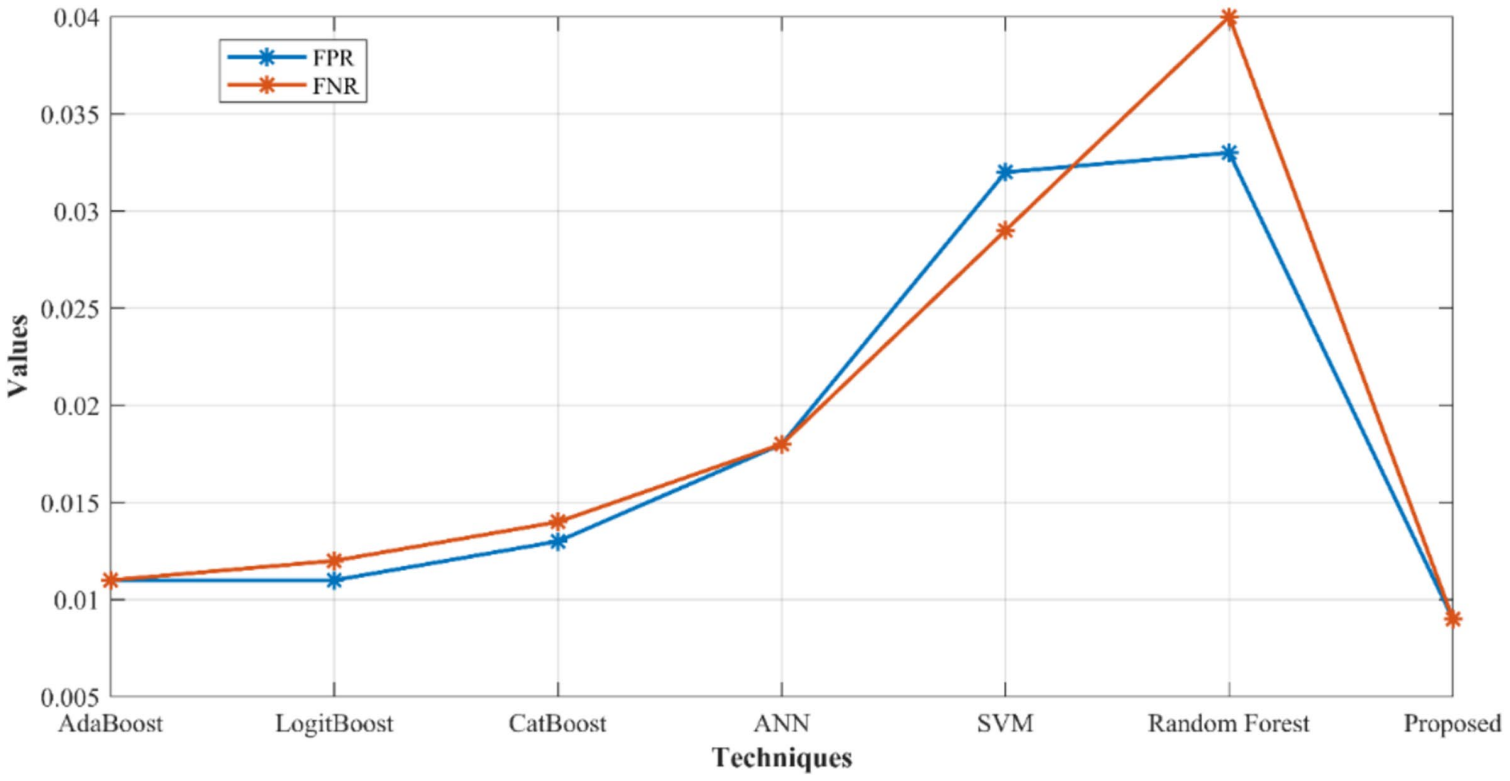
FPR and FNR analysis of proposed network over other networks.

Figure 5 specifies the performance of Positive Predictive Value (PPV), F-measure, Negative Predictive Value (NPV), and Matthew Correlation Coefficient (MCC) of developed model over other networks. Here, the developed model gets 0.2% better than AdaBoost, 0.4% better than LogitBoost, 0.6% better than CatBoost, 0.8% improved than ANN, 1.01% better than SVM, and 3.77% improved than RF for PPV. For F-measure, the proposed network reached 0.72, 0.51, 0.11, 0.21, 1.33, and 1.64% better than AdaBoost, LogitBoost, CatBoost, ANN, SVM and RF, respectively. Similarly, the developed model gets better results for NPV and MCC as well. In Figure 6, the False Positive Rates (FPR) and False Negative Rates (FNR) values are given. Here, the suggested model achieved enhanced results by outruns the existing models.

**Figure 7:**
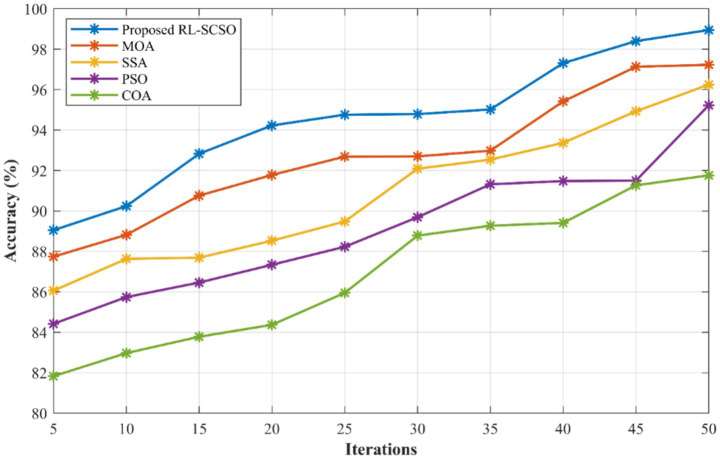
Convergence graph of proposed RL-SCSO model over other algorithms.

**Figure 8:**
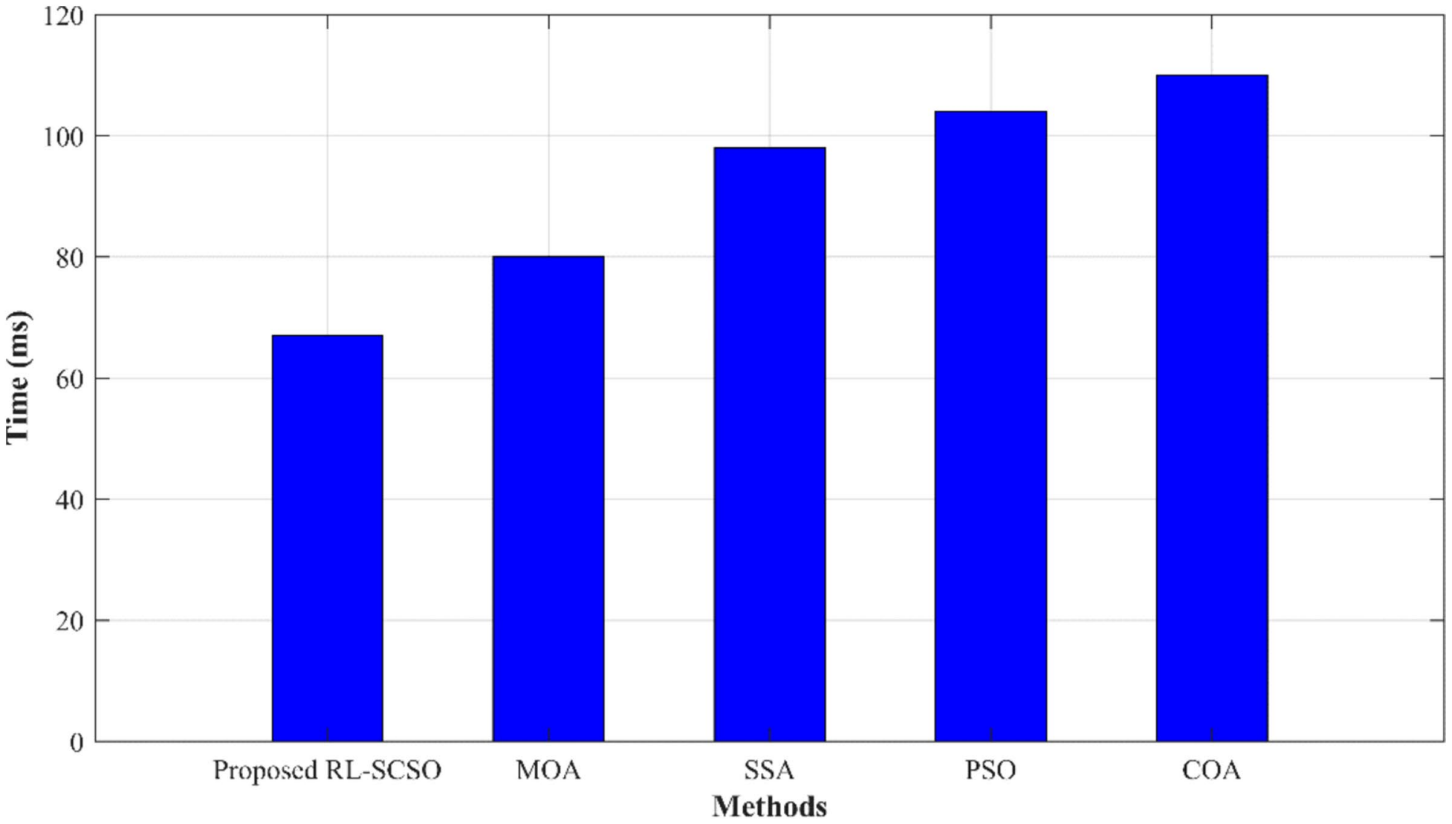
Time complexity of proposed RL-SCSO model over other algorithms.

Figure 7 shows the convergence of proposed RL-SCSO algorithm over other SOTA models. Here, the proposed one achieved better results which is 6.5% better than COA, 3.45% better than PSO, 2.34% better than SSA, 1.62% and improved than MOA. From Figure 7, it is evident that the proposed algorithm is competent in achieving better performance. Figure 8 shows the time complexity of proposed RL-SCSO model over other models. Hither, the proposed RL-SCSO obtained 1.03 (s) for selecting the optimal features which is 29.3, 58.06, 69.3, and 77.4% better than COA, PSO, SSA, and MOA, respectively (Table 1).

**Table 1:** Recent trends and technologies involved in CVD classification and security purposes.

Authors	Methods	Dataset	Weakness	Results
Konstantonis et al. (2022)	RF, SVM and LDA	CVD dataset	Failed to predict mild and early levels	Accuracy of 98% and AUC of 0.98
Kallimani et al. (2022)	ACNN-LSTM	Cleveland UCI Repository	Limited data were used for evaluation	Accuracy of 97%
Alqahtani et al. (2022)	ML Ensemble Model	CVD dataset	Need to improve the developed network performance	Accuracy of 88%
de Vries et al. (2023)	ANN	Cleveland Heart Disease dataset	Need to improve the performance better	Accuracy of 71% and sensitivity of 63%
Al Bataineh and Manacek (2022)	MLP-PSO Model	Cleveland Heart Disease dataset	Further improvement is mandatory concerning performance	Accuracy of 84%
Islam et al. (2023)	CNN	ECG Heartbeat Categorization Dataset - MITBIH Arrhythmia Database	Limited data source and sensor were used	Accuracy of 98% and F1-Score of 98%
Sadad et al. (2022)	DT, NB, SVM and 1D CNN-LSTM models	PPG-BP dataset	Exposed computational complexity	Accuracy of 99%
Samuel et al. (2023)	Blockchain-based Coalition Network	CVD dataset	Proof-of-work need to be improved	15% minimized computational cost and 26% proof-of-work
Rani et al. (2022)	ResNet50, VGG19, InceptionV3, and SqueezeNet	CVD dataset	Accuracy of the system is needed to be enhanced	Accuracy of 92%

**Figure 9:**
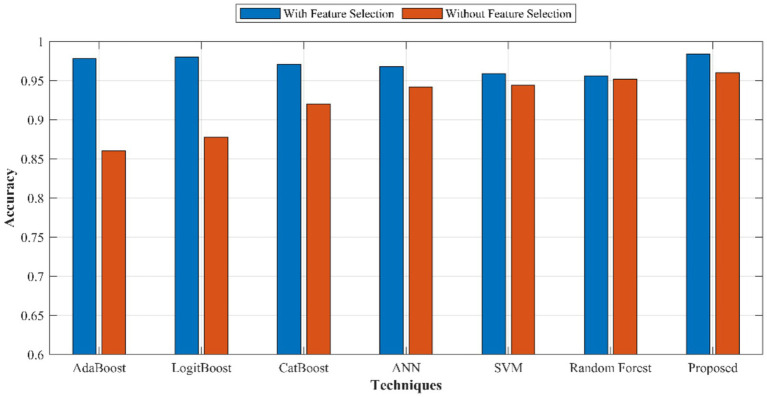
Performance of proposed Triboost ensemble model and other models with and without optimal feature selection.

From Figure 9, it is clear that the proposed RL-SCSO played a crucial role in attaining better performance. Also, the proposed RL-SCSO outruns the existing models. Thus, the optimal feature selection using proposed RL-SCSO showed its competence and efficiency in accomplishing better performance. The chosen baseline models that compared are ANN, SVM, RF, AdaBoost, LogitBoost and CatBoost, which are the most commonly used algorithmic families in CVD prediction, both classical machine learning classifiers and neural network-based methods and boosting ensembles. Such models have been widely used in the CVD literature and offer a reasonable description of conventional and ensemble learning strategies. Despite other boosting algorithms like XGBoost and LightGBM being more popular in general tabular classification problems, CatBoost was selected as the representative gradient boosting benchmark, because it has better capabilities to work with categorical clinical data, less hyperparameter optimization is required, and it is more computationally efficient in an IoT-blockchain setting.

Table 2 presents overall performance analysis of proposed model over other models. Here, the proposed model attained better accuracy of 98.4% which is higher than the existing models. Similarly, for sensitivity, specificity, and precision, the proposed model outruns other models. The proposed model has a high NPV of 0.991 and a MCC of 0.974, indicating its exceptional ability to accurately identify individuals without CVD while maintaining balanced sensitivity and specificity. Existing methods such as CatBoost, AdaBoost, LogitBoost, ANN, SVM, and RF also demonstrate strong performance but with slightly lower NPV and MCC values. Although these existing methods perform well, they fall slightly short compared to the proposed model.

**Table 2:** Overall performance analysis of proposed model over existing models.

Methods	Sen	Spec	Acc	Precision	PPV	F1-Score	NPV	FPR	FNR	MCC
AdaBoost	0.961	0.975	0.978	0.97	0.978	0.965	0.988	0.011	0.011	0.969
LogitBoost	0.96	0.981	0.98	0.97	0.976	0.967	0.986	0.011	0.012	0.97
CatBoost	0.963	0.973	0.971	0.97	0.974	0.971	0.978	0.013	0.014	0.963
ANN	0.96	0.962	0.968	0.968	0.972	0.97	0.968	0.018	0.018	0.954
SVM	0.956	0.951	0.959	0.966	0.97	0.959	0.956	0.032	0.029	0.934
RF	0.952	0.95	0.956	0.951	0.943	0.956	0.944	0.033	0.04	0.905
Proposed	0.969	0.988	0.984	0.975	0.98	0.972	0.991	0.009	0.009	0.974

**Figure 10:**
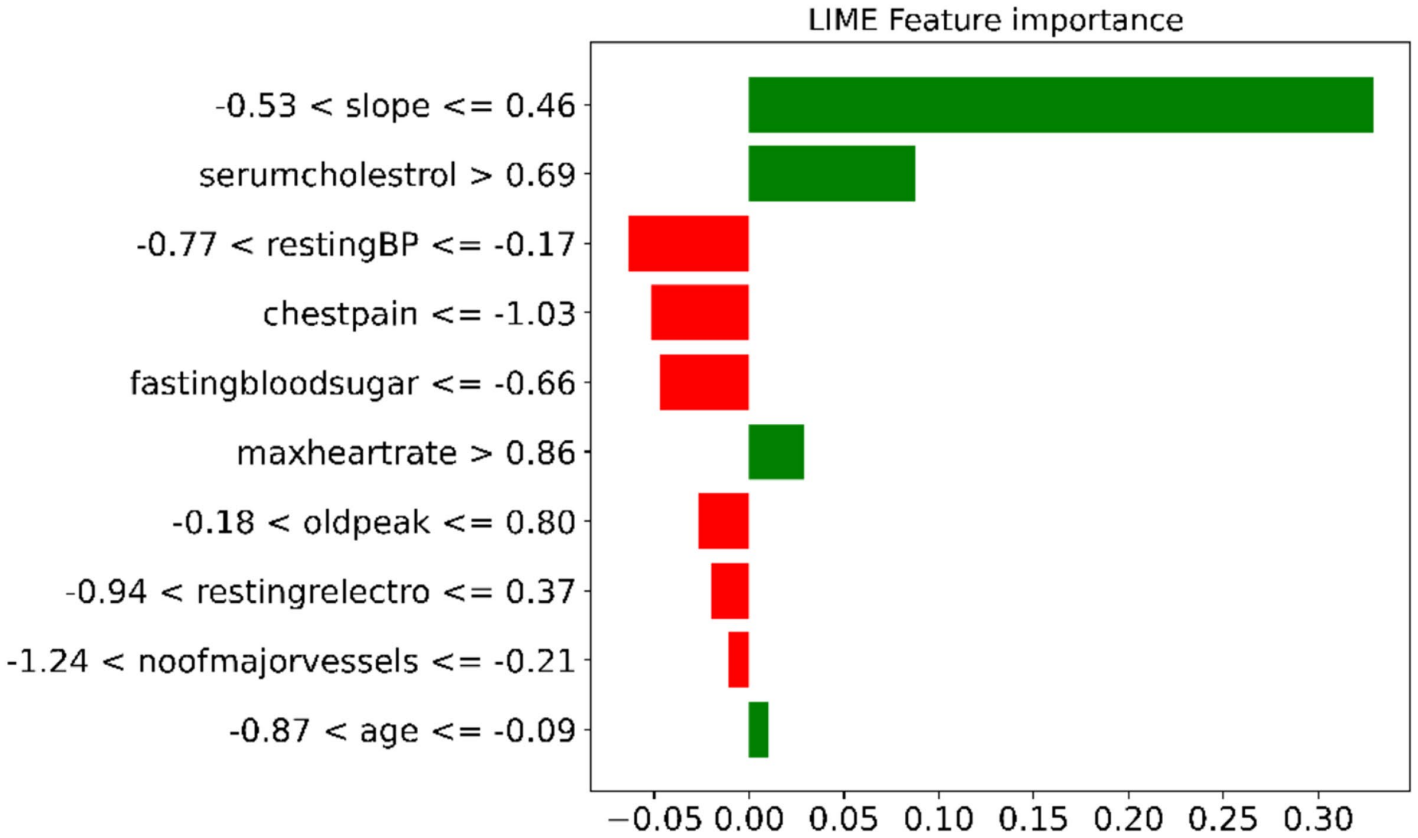
LIME feature-importance analysis.

Figure 10 shows the LIME feature-importance analysis, highlighting which clinical variables influenced the model’s prediction for a given case. Green bars indicate features that increase the likelihood of CVD, while red bars decrease it. The ST-segment slope and serum cholesterol have the strongest positive influence, whereas resting blood pressure, chest pain, and fasting blood sugar reduce the predicted risk. This demonstrates that the model relies on clinically meaningful features and provides interpretable decision insights.

**Figure 11:**
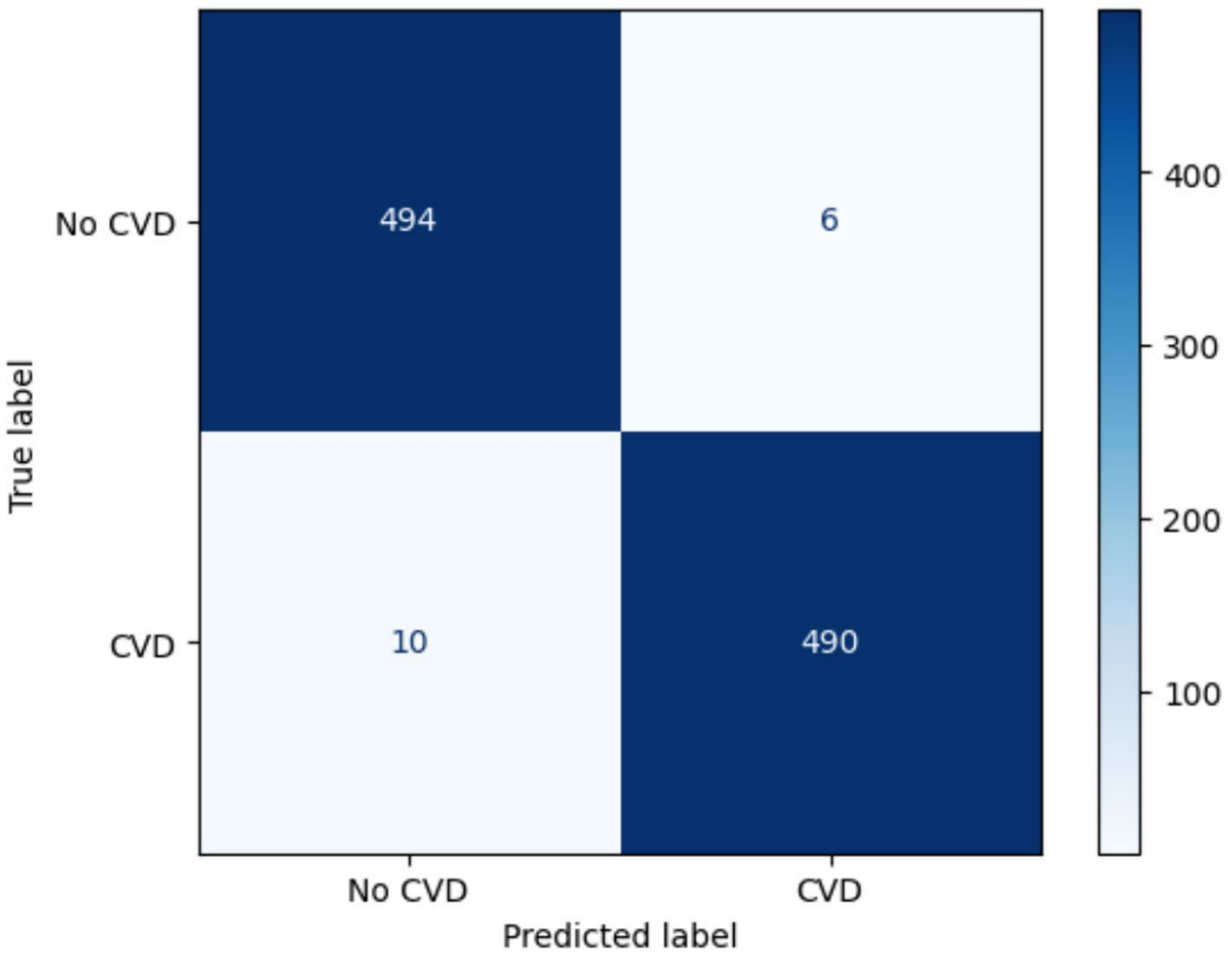
Confusion matrix of the proposed TriBoostCardio model.

Figure 11 illustrates the classification performance of the proposed TriBoostCardio model for distinguishing between CVD and non-CVD cases. The model correctly identified 494 out of 500 non-CVD samples and 490 out of 500 CVD samples, leading to very low false positives (6) and false negatives (10). The high number of correctly classified cases in both classes reflects the strong discriminative capability of the ensemble model. These results support the model’s high overall accuracy of 98.4% and demonstrate its effectiveness in reducing misclassification errors in practical CVD prediction scenarios.

**Figure 12:**
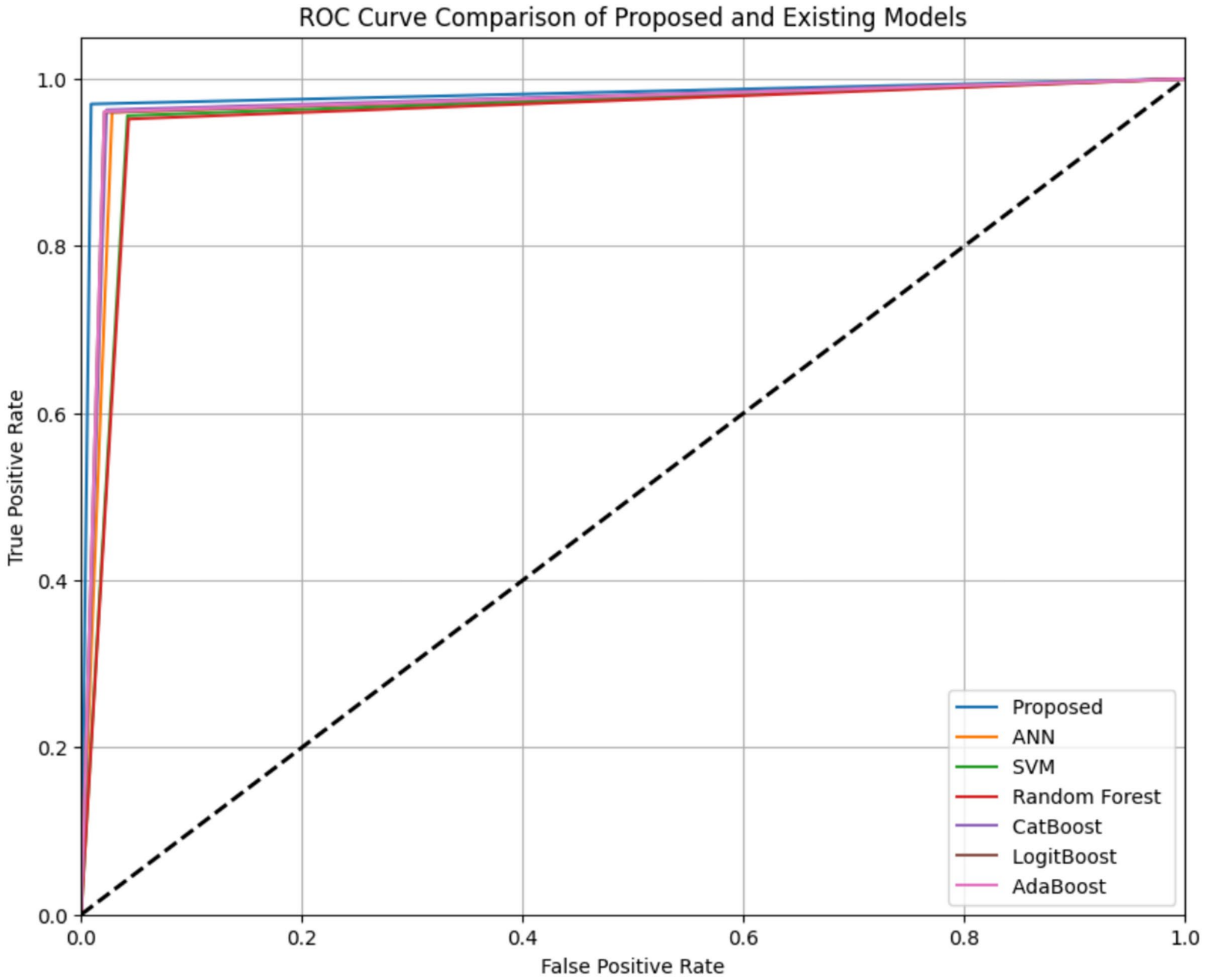
ROC curve comparison of the proposed TriBoostCardio model.

Figure 12 shows the ROC analysis for both the proposed TriBoostCardio model and the baseline classifiers. The proposed model achieves the highest AUC (0.984) with the lowest FPR (0.009), demonstrating its strong ability to separate CVD from non-CVD classes. The ROC curves of ANN, SVM, RF, CatBoost, LogitBoost, and AdaBoost show comparatively lower AUC values, confirming the superior diagnostic performance of the proposed ensemble.

### 4.2 Blockchain performance analysis

A controlled Ethereum test environment is used to assess the performance of blockchain. Storing a record took an average of 1.9 s to complete the transaction confirmation time, whereas authorization through smart-contract reads took less than 10 ms. The storeRecord function used around 120, 000 gas per transaction and the system had a throughput of 0.5 transactions per second when tested. The findings affirm that blockchain layer offers secure data processing with reasonable latency and calculation overhead to healthcare monitoring applications.

### 4.3 Privacy and security evaluation of the Blockchain layer

To ensure that the blockchain layer is effective in securing the data of the patients, a privacy and security assessment are performed. A test case is conducted whereby authorized clinicians and unauthorized users tried to access stored cardiovascular records. The access control based on smart contracts is able to allow all of the authorized and reject all of the unauthorized ones. The authorization function of the contract had a response time of 10 ms on average, which means that it had a small overhead in the process of retrieving secure data. These findings indicate that the blockchain aspect offers high privacy protection, high tampering resistance, and high access control to the suggested healthcare monitoring system.

### 4.4 Discussion

This research aims to introduce a novel technique to detect CVD in IoT settings. For data security, a blockchain mechanism is involved. Thereby, this research intends to achieve accuracy, security and efficiency. For this reason, an ensemble technique is utilized to detect the CVD and blockchain-enabled decentralized network is employed to ensure security and optimization concept is used to attain efficiency of the developed classifier by feeding it with optimal features. From this experimentation setup, the proposed TriBoostCardio ensemble model accomplished all the research goals such as enhanced accuracy 98.4%, security and efficiency. The efficiency of the proposed CVD detection framework is measured through a comparative study with the baseline and SOTA models.

The baseline methods such as CatBoost, AdaBoost, and LogitBoost models attained considerable performance however, medical data needs to be assessed carefully so that misclassifications are avoided. The proposed TriBoostCardio ensemble model outruns the baseline models by 0.6% better than AdaBoost, 0.4% better than CatBoost, and 1.32% better than LogitBoost. On the other hand, TriBoostCardio ensemble model accomplished better accuracy which is 1.62% improved than ANN, 2.54% improved than SVM, and 2.84% better than RF. Thus, the proposed TriBoostCardio ensemble model outperformed SOTA models and proved its efficiency in detecting CVD.

In addition to the improvement of performance, the suggested framework has significant clinical implications. TriBoostCardio model is added to the existing hospital information systems to facilitate real-time cardiovascular monitoring. The wearable IoT devices are capable of transmitting ECG, heart rate, and physiological data in real-time to hospital dashboards, which will clinicians monitor abnormalities sooner and intervene before complications occur. The access control system is also based on the blockchain that guarantees that the patient data shared among cardiologists, nurses, and emergency teams cannot be altered and is auditable to enhance confidence in digital health processes. Remote cardiac monitoring programs also be supported by the system, which means that high-risk patients is monitored at home with the smart wearables and avoid unnecessary visits to the hospital, which also allow receiving medical assistance in time. Such practical integration pathways underscore the fact that the model is implemented in the real clinical setting.

This research utilized open access datasets for experimentation which is limited due to certain reasons. Subsequently, real-time data using IoT wearables are sensitive and have private information of concerning patients. Acquiring these data is highly confidential and large volumes of data is difficult to gather. These constraints limit the data acquisition. Additionally, implementing blockchain technology in IoT settings requires high computational power and resources. Blockchain networks need a lot of energy, which is expensive, especially when they use proof-of-work consensus processes. On considering computational complexity, in future, simplified architecture will be designed with the use of various CNN architectures and real-time data will be gathered and train the network to achieve real-time applications for CVD detection in IoT settings.

## 5 Conclusion

In this paper, an advanced framework was developed for cardiovascular disease detection. The systematic process flow, from patient registration to data analysis, incorporates advanced features such as Refracted SCSO optimization for feature selection and the TriBoostCardio Ensemble Detection Model, ensuring a thorough and accurate examination of physiological data. The integration of smart contracts for access control enhances data security and privacy. Preliminary results showcase the effectiveness of the ensemble model, with an impressive overall accuracy of 98.4%. The model exhibits high sensitivity at 98%, ensuring a low rate of false negatives in identifying potential cardiovascular issues. The Negative Predictive Value (NPV) is at 99.1%, underscoring the model’s reliability in correctly identifying disease-free cases. The MCC stands at 97.4%, emphasizing the robustness of the ensemble model in capturing true positive and true negative cases. While experimental results show promising advancements in predictive accuracy, sensitivity, and specificity, further validation and refinement are imperative to assess the methodology’s robustness across diverse healthcare scenarios.

## Data Availability

The raw data supporting the conclusions of this article will be made available by the authors, without undue reservation.
